# Cereal Aphid Parasitoids in Europe (Hymenoptera: Braconidae: Aphidiinae): Taxonomy, Biodiversity, and Ecology

**DOI:** 10.3390/insects13121142

**Published:** 2022-12-12

**Authors:** Željko Tomanović, Nickolas G. Kavallieratos, Zhengpei Ye, Erifili P. Nika, Andjeljko Petrović, Ines M. G. Vollhardt, Christoph Vorburger

**Affiliations:** 1 Faculty of Biology, Institute of Zoology, University of Belgrade, 16 Studentski trg, 11000 Belgrade, Serbia; 2 Serbian Academy of Sciences and Arts, Knez Mihailova 35, 11000 Belgrade, Serbia; 3 Laboratory of Agricultural Zoology and Entomology, Department of Crop Science, Agricultural University of Athens, 75 Iera Odos Str., 11855 Athens, Greece; 4 Environment and Plant Protection Institute, Chinese Academy of Tropical Agricultural Sciences, Xueyuan Road 4, Haikou 571101, China; 5 Agroecology, Department of Crop Science, Georg-August University Göttingen, Grisebachstrasse 6, 37077 Göttingen, Germany; 6 Eawag, Swiss Federal Institute of Aquatic Science and Technology, Überlandstrasse 133, 8600 Dübendorf, Switzerland; 7 Institute of Integrative Biology, Department of Environmental Systems Science, ETH Zürich, 8092 Zürich, Switzerland

**Keywords:** integrated taxonomy, secondary endosymbionts, landscape complexity, trophic levels, hymenopteran parasitoids

## Abstract

**Simple Summary:**

Here, we review the current knowledge on the taxonomy, biodiversity, and ecology of cereal aphid parasitoids in Europe, which include 26 cereal aphid primary parasitoids and 28 hyperparasitoids. We present approaches to studying aphid–parasitoid–hyperparasitoid food webs, as well as the secondary endosymbionts in cereal aphids that may influence parasitoid community composition via their effects on food webs. We also review the effects of agricultural practices, environmental variation, and landscape complexity, on cereal aphid food webs and biological control.

**Abstract:**

Cereals are very common and widespread crops in Europe. Aphids are a diverse group of herbivorous pests on cereals and one of the most important limiting factors of cereal production. Here, we present an overview of knowledge about the taxonomy, biodiversity, and ecology of cereal aphid parasitoids in Europe, an important group of natural enemies contributing to cereal aphid control. We review the knowledge obtained from the integrative taxonomy of 26 cereal aphid primary parasitoid species, including two allochthonous species (*Lysiphlebus testaceipes* and *Trioxys sunnysidensis*) and two recently described species (*Lipolexis labialis* and *Paralipsis brachycaudi*). We further review 28 hyperparasitoid species belonging to three hymenopteran superfamilies and four families (Ceraphronoidea: Megaspillidae; Chalcidoidea: Pteromalidae, Encyrtidae; Cynipoidea: Figitidae). We also compile knowledge on the presence of secondary endosymbionts in cereal aphids, as these are expected to influence the community composition and biocontrol efficiency of cereal aphid parasitoids. To study aphid–parasitoid–hyperparasitoid food webs more effectively, we present two kinds of DNA-based approach: (i) diagnostic PCR (mainly multiplex PCR), and (ii) DNA sequence-based methods. Finally, we also review the effects of landscape complexity on the different trophic levels in the food webs of cereal aphids and their associated parasitoids, as well as the impacts of agricultural practices and environmental variation.

## 1. Introduction

Cereals dominate in the European agricultural landscape, by encompassing almost one-third of the European agricultural area [1]. It is well known that aphids are one of the most important limiting factors of cereal production, through direct damage or by transmitting viral pathogens (e.g., barley yellow dwarf virus, wheat dwarf virus) [2,3,4,5,6,7]. Aphid parasitoids are diverse and effective natural enemies of cereal aphids [8,9,10,11,12,13,14,15,16]. It should be noted that the high importance of aphidiines as natural enemies of cereal aphids led to their introduction into North and South America [17,18,19] and Australia [20,21] to control cereal aphids.

Brewer and Elliott [19] reported classical biological control efforts against cereal aphids in North America using parasitoids and predators. Cereal aphids and their parasitoids are common models for numerous ecological and evolutionary studies focusing on tri-trophic food web interactions (cereal aphid-parasitoid-hyperparasitoid) [13,22,23,24,25]; landscape effects on parasitoid species diversity [26] or biological control [13,27,28]; and the importance of agricultural practices on parasitoid effectiveness in cereal agroecosystems [24,29,30]. Most studies about cereal aphids and related parasitoids consider a few economically important species, usually at a local or regional scale [12,31,32,33,34]. Hyperparasitoids have the potential to compromise biological control by primary parasitoids [35,36]. The species composition of hyperparasitoids, as a top consumer in cereal aphid–primary parasitoid systems, has not been sufficiently explored. For instance, Tomanović et al. [12] reported the relative abundance of several hyperparasitoid species feeding on aphidiines attacking four major cereal aphid species in Serbia. Ye et al. [37] explored 16 species of hyperparasitoids from European cereal crops. Surprisingly, the basic biological and taxonomic characteristics of species participating in cereal aphid food webs are poorly known, including the cereal aphids’ endosymbionts, potentially affecting parasitoids and hyperparasitoids [38]. Starý [9] and Kavallieratos et al. [10] reviewed parasitoid associations and provided keys for the identification of cereal aphid parasitoids in the western Palaearctic and Southeastern Europe, respectively. Several studies have presented the knowledge to date about cereal aphids and their natural enemies in Europe [2,10,12,39,40]. However, there have been no updates on the biodiversity, taxonomy, and ecology of cereal aphid parasitoids and their endosymbionts. Therefore, the aim of the current review was to present knowledge about the taxonomy of cereal aphid parasitoids; species composition, including allochthonous parasitoids, food webs, and the effects of agricultural practices and landscapes on parasitoid diversity and efficiency; and the role of endosymbionts in biological control.

## 2. Cereal Aphids in Europe

Aphids are common pests in European cereal crops. Species from the genera *Sitobion* (*Sitobion avenae* (F.)), *Metopolophium* (*Metopolophium dirhodum* (Walker)), and *Rhopalosiphum* (*Rhopalosiphum padi* (L.), *Rhopalosiphum maidis* (Fitch)) are the most common on wheat, barley, maize, and oat crops in Europe [40,41]. In addition, climate change leads to the increasing pest importance of *Schizaphis graminum* (Rondani) in European cereal crops [42]. According to Blackman and Eastop [3], 34 aphid species are known to infest cereal crops in Europe. Some of those that are attacked by primary parasitoids and hyperparasitoids are mentioned in Table 1 and Table 2.

## 3. Primary Parasitoid Species Identification and Their Taxonomy

Identification is based on morphological characters. Some cereal aphid parasitoids are taxonomically well-defined and identifiable morphologically with existing keys for the identification of species [9,10,31,56]. Based on current records, 13 Aphidiinae genera include species that attack cereal aphids in European cereal agroecosystems. 

### 3.1. Aphidius Nees

Across Europe, species from the genus *Aphidius* are the dominant parasitoids of different cereal aphids [10,32,33,57,58]. Among them, *Aphidius rhopalosiphi* De Stefani and *Aphidius uzbekistanicus* Luzhetzki are key species for the biological control of cereal aphids. They are also taxonomically the most problematic species [9,59,60]. According to Eady [61], both species belong to a group with a costulate anterolateral part of the petiole. Based on morphological characteristics, their identification often leads to misidentification. Several papers have considered the separation and characterization of both species [9,59,60,62], but due to their overlapping morphological characters, there is a list of synonyms of these species [9,56]. Summarizing all previous research efforts on the taxonomy *A. rhopalosiphi* and *A. uzbekistanicus*, the two species can be distinguished on the basis of the following characteristics: *A. uzbekistanicus* has a broad triangular forewing pterostigma, a narrow yellow ring at the base of flagellomere I, and usually fewer antennal segments (15–16); while *A. rhopalosiphi* has a more elongated pterostigma, a variable color pattern of flagellomere 1 and 2 (with predominantly yellow parts of flagellomere 1 and occasionally flagellomere 2), and usually 16–17 segmented antennae [60]. *Aphidius matricariae* Haliday represents an additional species with a costulate petiole and an elongated pterostigma, but it is clearly characterized by three-segmented maxillary palps and two-segmented labial palps, while those of *A. rhopalosiphi* and *A. uzbekistanicus* are four- and three-segmented, respectively [63,64]. *Aphidius avenae* Haliday and *Aphidius colemani* Viereck belong to the group with a costate anterolateral area of the petiole, but are clearly distinguished by shallow costae and the light-colored body of *A. colemani*, in contrast to the deep ridges on the anterolateral part of the petiole and a dark-colored body of *A. avenae* [63,65]. Additionally, the number of antennal segments in *A. colemani* is 14–15 vs. (16) 17–18 in *A. avenae* [11,63,66]. Although *Aphidius picipes* (Nees) is a commonly used name, it is a synonym of *A. avenae*. *Aphidius ervi* Haliday is an abundant cereal aphid parasitoid and the most important parasitoid of *Acyrthosiphon pisum* (Harris) in European legumes [67]. This is a large species, belonging to the group with a rugose anterolateral area of the petiole, and it is clearly separated from other congeners in cereal aphid parasitoid communities [10].

### 3.2. Praon Haliday

Four *Praon* species are known to be members of the cereal aphid parasitoid community in Europe. These species are divided into two groups, with either a developed forewing medio-cubital vein (m + cu) (*Praon volucre* (Haliday) and *Praon abjectum* (Haliday)) or with an effaced m + cu vein (*Praon gallicum* Starý and *Praon necans* Mackauer). *Praon volucre* is the most common *Praon* species in cereal agroecosystems in Europe and easily recognizable, with (16)17–18(19) segmented antennae, while *P. abjectum* has 14–15 segmented antennae. *Praon gallicum* is generally yellow to light brown, with yellow F1 and F2, while *P. necans* is generally darker with brown F1 and F2. For more details about morphological identification see Starý [9], Kavallieratos et al. [68] and Rakhshani et al. [69].

### 3.3. Binodoxys Mackauer

From this genus, only *Binodoxys angelicae* (Haliday) parasitizes cereal aphids in Europe. All species from this genus are characterized by possessing two prongs on the last abdominal sternite for host grasping, and two pairs of tubercles on the petiole [9]. For more details about the taxonomy of *Binodoxys* see Lazarević et al. [70].

### 3.4. Trioxys Haliday

Two *Trioxys* species are known to parasitize cereal aphids in Europe: *Trioxys auctus* (Haliday) and the North American species *Trioxys sunnysidensis* Fulbright and Pike. Both species share common characteristics, such as 12 segmented antennae, 4–6 setae on dorsal prongs, and two simple bristles on the top of prongs, but they are easily distinguished by the numerous longitudinal striations on the dorsal side of the petiole of *T. auctus* [71,72].

### 3.5. Lysiphlebus Förster

There are three *Lysiphlebus* species on cereal aphids in Europe (*Lysiphlebus fabarum* (Haliday)*, Lysiphlebus testaceipes* (Cresson), and *Lysiphlebus dissolutus* (Nees) on root aphids—see below). *Lysiphlebus fabarum* is a native European species, characterized by a long forewing R1 vein (= metacarpus), thickened antennae, and setae on the forewing edge that are shorter than those on the forewing surface. This species occurs in both sexual and asexual populations [73]. The allochthonous *L. testaceipes* (Nearctic species) has a short R1 vein and long setae along the forewing edge.

### 3.6. Ephedrus Haliday

*Ephedrus* species have braconid-like wing venation patterns and 11 antennomeres in both sexes. Only *Ephedrus plagiator* (Nees) is a member of the cereal aphid parasitoid community in Europe, while *Ephedrus persicae* Froggatt attacks some aphids using cereals as secondary host plants, but only on their primary host plants [9]. *Ephedrus plagiator* has a longer three-SR vein than two-SR and a more elongated petiole than *E. persicae* [9,74,75].

### 3.7. Lipolexis Förster

Although *Lipolexis gracilis* Förster has been considered the only member of the genus *Lipolexis* attacking cereal aphids (*R. padi*) [9], Kocić et al. [46] recently identified an additional species, *Lipolexis labialis* Tomanović and Kocić, which parasitizes *Anoecia corni* (F.) on wheat. The genus is characterized by needle-like ovipositor sheaths and reduced wing venation. The two species can be separated from each other by the number of labial palpomeres (two in *L. labialis* and one in *L. gracilis*) and by a more elongated first flagellomere in *L. labialis*.

### 3.8. Additional Parasitoid Species in Cereal Fields across Europe

*Diaeretiella rapae* (M’Intosh) is an easily recognizable species with (13)14 segmented antennae, narrow areola on the dorsal side of propodeum, reduced wing venation pattern, and a R1 vein that is shorter than the pterostigma [10,76,77]. *Monoctonus caricis* (Haliday) is the only *Monoctonus* species parasitizing cereal aphids. It has ploughshare shaped ovipositor sheaths and 13 segmented filiform antennae [47]. *Adialytus ambiguus* (Haliday) is a specialized parasitoid of *Sipha* spp. aphids on cereals in Europe. Morphologically characterized by a reduced forewing venation with radial vein and R1 vein longer than the pterostigma. Its propodeum bears two divergent carinae at the base [43,78].

*Toxares deltiger* (Haliday) is the only known species of the genus *Toxares* in Europe, and it is characterized by a braconid-like wing venation pattern, deltoid shaped ovipositor sheaths and antennae with 18–20 antennomers [79].

A specific aphid parasitoid fauna is present on cereal root aphids, i.e., *Aclitus obscuripennis* Förster (marginal cell of the forewing closed or with narrow opening, 15 segmented antennae, ovipositor sheaths short and triangular), *L. dissolutus* (15–16-segmented antennae with subsquare flagellomeres and carinated propodeum), and *Paralipsis enervis* (Nees) (reduced wing venation with a very short triangular pterostigma without R1 vein [73,80,81]. These parasitoids exhibit very specific morphological peculiarities (small eyes, square antennal segments, short body, and strong legs), due to adaptation to their specific ecological niche.

## 4. Integrative Taxonomy

Although some species of cereal aphid parasitoids are morphologically well-defined, the identification and taxonomy of others are still very complicated and require an integrative approach. Over the last decade, researchers have incorporated molecular methods as additional tools, together with morphology and ecology, for the accurate identification of aphidiine wasps [82]. Cereal aphid parasitoids belonging to the genus *Aphidius* are among the first Aphidiinae whose molecular data (cytochrome *c* oxidase subunit I (COI) sequences) were used to determine their taxonomic status. Kos et al. [60] used morphological data (e.g., traditional and geometric morphometrics), molecular data (COI sequences), and ecological data (trophic relationships) to resolve the taxonomic status of *A. uzbekistanicus*, *A. rhopalosiphi*, and the American species *A. avenaphis* (Fitch). The authors found very distinct mitochondrial lineages within several *A. rhopalosiphi* populations, correlated with morphological variability, and indicating the existence of cryptic species within this taxon.

Derocles et al. [83] confirmed the utility of COI sequences as a genetic marker for Aphidiinae identification and also presented their limitations for sole use. After these studies, an integrative approach became the “gold standard” in Aphidiinae taxonomy [82], which resulted in numerous and interesting new findings in cereal aphid parasitoids. For example, by combining the mitochondrial COI (cytochrome c oxidase subunit I) barcoding gene and geometric morphometrics, Mitrovski-Bogdanović et al. [84] found that *P. abjectum* represents a complex of sibling species. It was determined that *P. abjectum*, similarly to wasps parasitizing *Aphis sambuci* L. on common elder (*Sambucus nigra*), a very common plant in seminatural habitats in cereal fields, represents a different species named *Praon sambuci* Tomanović and Starý. Besides genetic and ecological differences, *P. sambuci* has morphological traits that separate it from *P. abjectum*, such as a broader pterostigma. Furthermore, the use of an integrative approach in studies of cereal aphid parasitoids led to the discovery of previously undiscovered allochthonous species and of species that were new to science.

## 5. Allochthonous Parasitoid Species

Two allochthonous species of cereal aphid parasitoids, whose role in multitrophic food webs is still poorly known, appear in European cereal agroecosystems. *Lysiphlebus testaceipes* was introduced from Cuba to southern France in 1973, for the control of citrus aphids [85]. However, this parasitoid attacked numerous aphid hosts, including cereal aphids, in this new environment [10,34,44,86] and spread along the Mediterranean coast, also colonizing inland habitats [87,88,89]. *Trioxys sunnysidensis* has been described from the USA in association with *R. padi* infesting wheat near irrigation channels [71]. The authors found *T. sunnysidensis* in open wheat fields in Germany attacking *R. padi* [26,50]. Due to its morphological similarity with *T. auctus* and probably also *B. angelicae*, this species has been overlooked for a long time in European cereal agroecosystems; therefore, all former records need to be revisited for a thorough re-examination. Although we considered this species as allochthonous and probably unintentionally introduced from North America to Europe, our findings on haplotype diversity of *T. sunnysidensis* imply a possible European origin and Holarctic distribution [50].

## 6. New Aphid Parasitoids in European Cereal Agroecosystems

Two new species of cereal aphid parasitoids have been described in the last couple of years. The previously mentioned *L. labialis* was newly described as a parasitoid of *A. corni* and has been found in several European countries [46]. *Paralipsis* spp. parasitize root aphids [80,81]. Little is known about the economic effects of root aphids on cereals [90], even though they exhibit considerable diversity in European cereal fields [91]. *Paralipsis enervis* is a specialized parasitoid of root aphids that has been recorded on *Geoica utricularia* (Passerini) [44]. After careful examination of many *Paralipsis* specimens, a new sibling species was described from this genus, *Paralipsis brachycaudi* Tomanović and Starý, emerging from *Tetraneura ulmi* (L.) [48].

## 7. Species Complexes

Cryptic speciation in aphid parasitoids is a very common phenomenon. Especially within what had seemed to be oligophagous species exhibiting broad host ranges, some host associated lineages had to be newly described as cryptic species, after applying modern integrated taxonomic approaches [73,92]. Additionally, it seems that widely distributed generalist species often cover a narrower host range at a local scale, driven by trophic specialization and environmental factors in specific areas [93]. For example, the broadly oligophagous *D. rapae* attacks only a few aphid hosts in the continental part of Southeastern Europe, mainly *Brevicoryne brassicae* (L.) in the lowlands, and exceptionally *Myzus persicae* (Sulzer) under glasshouse conditions. Additionally, *D. rapae* parasitizes some specific non-crop aphids occurring in the mountains of Southeastern Europe (e.g., *Aphis cadiva* Walker, *Hayhurstia atriplicis* (L.)*,* and *Pseudobrevicoryne leclanti* Petrović-Obradović and Remaudière) [44]. In contrast, the host range of this species in the Mediterranean is very diverse and composed of >20 aphid species, including some cereal aphids (*Diuraphis noxia* (Kurdjumov)*, R. padi, Rhopalosiphum maidis* (Fitch)) [44,45]. A second example is *A. avenae*, which is more frequently recorded as a member of the cereal parasitoid spectrum in Central and Western Europe than in Southern Europe, where it is more abundant in high mountains as a parasitoid of some *Sitobion* aphid species [44,63]. Therefore, it has become evident that local specialization plays an important role, since generalist species behave as specialists at local scale in broader geographic areas [93]. Further research is needed to confirm the status of host-associated lineages in broadly oligophagous parasitoid species.

Several authors have considered the taxonomic status of *A. rhopalosiphi* and *A. uzbekistanicus* as two sibling species (“*rhopalosiphi*-*uzbekistanicus*” species complex). Apart from their overlapping morphological characters, they also share a similar host range pattern (Table 1). Kos et al. [60] found that *A. uzbekistanicus* shows very restricted variation, both genetically and morphologically. In *A. rhopalosiphi*, on the other hand, several mitochondrial lineages exist, forming a highly diverse haplotype network [60]. The status of this large number of haplotypes should be re-evaluated in future research. Electromorph variability, host range, and breeding experiments indicated *A. ervi* as a species complex consisting of several sibling taxa [94]. Until now, only *Aphidius microlophii* Pennacchio and Tremblay has been split from *A. ervi* and described as a specialized parasitoid of *Microlophium carnosum* (Buckton), associated with *Urtica* spp., which are common in cultivated areas [95,96]. Molecular markers did not support any significant differentiation within the “*ervi*” complex [97]. This issue should be further investigated with more robust taxonomic and geographic samples. Mitrovski-Bogdanović et al. [84] demonstrated that *P. abjectum* represents a species complex and described *P. sambuci* and *Praon longicaudus* Tomanović and Starý. In addition, further research on the various *P. abjectum* biotypes (e.g., of *Aphis* spp. hosts) should be conducted. *Praon volucre* is a broadly oligophagous species attacking a wide spectrum of aphid hosts [98] and also exhibiting possible biotype diversification [92]. However, a previous investigation did not reveal any specific genetic or morphological lineages within several *P. volucre* biotypes [99]. Rakhshani et al. [78] recognized by revealing morphological diversification within *A. ambiguus,* “*Adialytus* cf. *ambiguus”* (Haliday) and “*Adialytus arvicola*” (Starý) emerging from *Sipha* aphid hosts. Both phenotypes required further taxonomic treatment.

## 8. Host Location, Specialization, and Exploitation of Aphid Colonies

Cereal aphids belong to several aphid tribes in Europe (i.e., Aphidini, Macrosiphini, Pterocommatini, Anoecini, Eriosomatini, Fordini) that are characterized by weak mobility, and feeding upon leaves, green stems, ears, or roots [100,101,102,103]. Aphid colonies are located on the open surface of cereals, except *D. noxia* which grows within curled leaves. All cereal aphids are medium-sized (except the small-sized *D. noxia*) and develop dense colonies. Most cereal aphids are monoecious and feed only on cereals and other Poaceae, except for *M. dirhodum* and *R. padi*, which are heteroecious. They migrate in autumn to primary woody host plants other than Poaceae, such as *Rosa* spp. and *Prunus padus*, respectively, where they mate and deposit their overwintering eggs [3]. It is well known that the most abundant and the most effective Aphidiinae parasitoids in European cereal agroecosystems are *A. rhopalosiphi, A. uzbekistanicus, A. ervi, P. volucre,* and *E. plagiator* [12,13,26,33,57,104], with the addition of *A. matricariae, D. rapae,* and allochtonous *L. testaceipes* in the Mediterranean area [10,34,44,45,105]. Since a measure of the efficiency of parasitoids in exploiting their hosts is their abundance on the hosts they attack [106,107], we can conclude that the most important cereal aphid parasitoid assemblages in Europe consist of oligophagous specialists (*A. uzbekistanicus, A. rhopalosiphi,* and *A. ervi*) and broadly oligophagous generalists (*P. volucre* and *E. plagiator*), with additional oligophagous generalists from the Mediterranean areas (*A. matricariae, D. rapae,* and *L. testaceipes*). According to Straub et al. [108], there are two hypotheses explaining the relationships between host-range breadth and the abundance of parasitoids. According to the resource–breadth hypothesis, generalist parasitoids are more abundant than specialists on common hosts, exhibiting an advantage under environmental instability (e.g., agroecosystems, urbanization) and low available resources. The trade-off hypothesis gives more advantages to specialists under a more stable environment [109]. In the case of cereal aphid parasitoids, a mixed complex of parasitoids (oligophagous specialists and oligophagous generalists) could provide adequate biological control and ecosystem stability through control of aphid populations [110].

According to van Baaren et al. [111,112], the cereal aphid parasitoid *A. rhopalosiphi* attacks *S. avenae*, as the most abundant parasitoid species present in the field prior to the arrival of *A. ervi* and *A. avenae*, by exploiting aphid colonies when they start to produce cornicle secretions. Upon arrival, *A. ervi* and *A. avenae* parasitize those aphid colonies that have not been exploited by *A. rhopalosiphi*.

There has been little research about the ability of cereal parasitoids to distinguish between parasitized and non-parasitized aphid hosts, an issue that is extremely important for the aphid host selection in parasitoids. Recognition of already parasitized aphid hosts by parasitoids is done using chemical cues in their cornicle secretion and exuviae, in combination with visual cues [113,114,115]. Outreman et al. [116] reported that *A. rhopalosiphi* recognizes already parasitized aphid hosts using external factors such as aphid alarm pheromone in cornicle secretion or by internal factors after the insertion of its ovipositor into the aphid’s body. However, these host discriminations are species-specific [117,118]. Van Baaren et al. [112] showed that *A. rhopalosiphi* recognizes *S. avenae* parasitized by *A. ervi* on the basis of cornicle secretion, which is released after parasitization. However, aphid hosts already parasitized by *A. avenae* do not alert others in the aphid colony [112]. The exploitation of aphid colonies by aphid parasitoids is mediated by many different factors, including host quality and the presence of different host defenses (e.g., mobility, wax production, and microorganisms) [119,120,121].

## 9. Primary Parasitoids and their Hyperparasitoids

Secondary parasitoids represent the fourth trophic level in multitrophic aphid–parasitoid communities and have the potential to exert top-down control in such food webs [122,123,124]. According to Sullivan and Völkl [123], there are several categories and subcategories of secondary parasitoids, of which two are relevant for aphidiines. “True” hyperparasitoids attack primary parasitoids at the larval stage within the living aphid and develop inside the primary parasitoid host, without arresting its development; hence, they are referred to as koinobiont endohyperparasitoids. Idiobiont ectohyperparasitoids, also referred to as mummy parasitoids, attack primary parasitoid prepupae and pupae, on which they develop externally within the empty aphid cuticule (the mummy). In Table 2, we review 28 hyperparasitoid species belonging to three hymenopteran superfamilies and four families (Ceraphronoidea: Megaspillidae; Chalcidoidea: Pteromalidae, Encyrtidae; Cynipoidea: Figitidae).

Generally, idiobiont ectohyperparasitoids on cereals have a broad host range, while koinobiont endohyperparasitoids exhibit host specificity, except for *Syrphophagus aphidivorus* (Mayr)*, Alloxysta brevis* (Thomson)*, Alloxysta victrix* Westwood, and *Phaenoglyphis villosa* (Hartig) (Table 2). However, cryptic speciation abounds in these wasps, especially in *Alloxysta* spp. [125], and field records of their aphid-primary parasitoid hosts are usually missing, such that the true extent of their host specificity remains elusive.

Hyperparasitoids disrupt biological control directly, by affecting primary parasitoids at variable levels of specialization [36,53,54,126] or as intraguild competitors (facultative hyperparasitoids) [127]. However, there is a huge debate about the role of hyperparasitoids as a fourth trophic level in the entire trophic systems. Some authors point out the positive role of hyperparasitoids in stabilizing the whole trophic chain through density-dependent interactions [126,128,129]. Due to higher thermal requirements and the time-lag inherent in consumer–resource relationships, hyperparasitoids appear later in the season in comparison with aphids and their primary parasitoids [36,122]. However, due to climate change and land-use changes through landscape simplification, higher temperatures could increase and positively affect the hyperparasitoid community, leading to pest increase [130]. Additionally, the increasing temperature causes primary parasitoids and hyperparasitoids to lose their diapause, an issue that negatively affects the abundance of primary parasitoids in the spring, due to the winter activities of hyperparasitoids [131,132]. However, the effects of climate change on the whole cereal aphid–parasitoid trophic system are still unpredictable and require further research.

## 10. Methods for Determining Trophic Interactions of Cereal Aphids and their Associated Parasitoids

Traditionally, the methods for determining trophic interactions among cereal aphids and their parasitoids have mainly included morphological approaches such as dissection and rearing. Direct host dissection provides an unequivocal estimate of parasitism rate, as well as multiparasitism, superparasitism, and hyperparasitism [55,133,134,135]. However, identifying the eggs or larvae of parasitoids inside their hosts is difficult and often impossible, due to a lack of distinguishable morphological characteristics [136,137]. On the other hand, the primary parasitoid and hyperparasitoid adults reared from field-collected hosts can be morphologically identified at the species level and linked directly with the aphid species they emerge from [138]. To link the hyperparasitoids to their real host (i.e., primary parasitoids), it is possible to identify the hyperparasitoid species and determine their host (i.e., primary parasitoids) at the genus level based on the morphological characteristics of aphid mummies [124,139]. Nevertheless, species-specific links between primary parasitoids and hyperparasitoids still remain obscure [140,141]. Additionally, rearing approaches are affected by delayed parasitoid emergence, as well as host and parasitoid mortality. There are also hurdles when facing multiparasitism and superparasitism [136,137].

Molecular approaches, mainly DNA-based approaches, can overcome these limitations [137]. They have been shown to allow for species-specific examination of trophic interactions between primary parasitoids (e.g., multiparasitism), as well as between primary and secondary parasitoids (e.g., hyperparasitism) [22,24,142]. These approaches remain unaffected by delayed parasitoid emergence, as well as host and parasitoid mortality, and can be applied to each developmental stage [136]. To date, there have been two kinds of DNA-based approaches used to determine aphid–parasitoid–hyperparasitoid food webs: (i) diagnostic PCR methods (mainly multiplex PCR), and (ii) DNA sequence-based methods [137]. Multiplex PCR allows the amplification of several targets in parallel within a single reaction [22,25,142,143], compared to singleplex PCR [144]. However, diagnostic PCR approaches require prior knowledge of all potential species in the study area [145,146], and might be affected by unexpected trophic interactions, such as alternative food sources and invasive species [147,148]. DNA sequence-based methods, on the other hand, can overcome these limitations and have been used to identify parasitoid DNA using universal primers, followed by Sanger sequencing [83] or next-generation sequencing (NGS) [149]. The issue with Sanger sequencing is that, when there are multiple species in the same DNA extract, it either obtains one DNA sequence or even sequencing failure, as in the case of hyperparasitim and multiparasitim [83]. The NGS approach can overcome this hurdle [150], but it is affected by different target DNA proportions among test samples [145]. Compared to rearing and morphological identification of parasitoids, another limitation of molecular approaches is the difficulty in estimating the superparasitism and real biocontrol efficiency of parasitoids, as they can detect the presence of parasitoid eggs and larvae within the host even when they would not have survived to complete their development [52,135,151].

## 11. Cereal Aphid–Primary Parasitoid–Hyperparasitoid Food Webs

Host–parasitoid food webs are among the most studied trophic interactions. They have been investigated as models to address important ecological questions, such as robustness and restoration of ecological networks [152], bottom-up effects and habitat effects on food web structures [153,154], as well as apparent competition [155,156].

The cereal aphid–primary parasitoid–hyperparasitoid food webs across different habitats have been broadly surveyed and analyzed [157,158]. These networks have also been investigated to understand important questions in trophic ecology, such as parasitoid host specificity [100,159], intraguild predation [160,161], and the effects of climate change on trophic networks [162,163,164]. Further studies were concerned with how farming practices affect these trophic interactions, including the effects of agriculture intensification [23,24,124,165], landscape changes [139,166], habitat modification [167,168], and pesticide application [169]. To date, several cereal aphid–primary parasitoid–hyperparasitoid food webs have been reconstructed in different geographic regions around the world [22,143], exhibiting partial spatiotemporal variation [159].

Aphid–primary parasitoid–hyperparasitoid networks are linked to other trophic levels, such as plants, plant pathogens, and predators, forming wider and much more complex trophic interaction networks. Plants can have cascading effects on aphid–primary parasitoid–hyperparasitoid food webs. For example, negative impacts of aphid resistant soybeans on the development, mummification, and adult parasitoid emergence have been observed [170]. Among plant-derived defense chemicals, beta-aminobutyric acid has a negative impact on the size of emerging *A. ervi* [171]. On the other hand, acetylsalicylic acid, oxalic acid, and glucosinolates can positively affect aphid parasitoids [172,173]. Genetically-modified crops seem not to have strong effects on the non-target pests, aphids and their associated parasitoids. For cereal, the impact of powdery mildew-resistant GM wheat on cereal aphid–parasitoid–hyperparasitoid food webs seems negligible and limited [174]. Moreover, in other crop systems, transgenic Cry1Ac + CpTI cotton cultivars do not affect aphid–parasitoid food webs and biological control [142]. Although transgenic potatoes with nematode resistance can reduce the density of aphids, no effect on higher trophic levels (i.e., primary- and hyperparasitoid) has been found [175].

Besides plant traits, plant-associated symbionts or pathogens can affect aphid–primary parasitoid–hyperparasitoid food webs. Plant protective symbiotic fungal infection can depress both aphids and parasitoids [176,177,178,179,180]. Arbuscular mycorrhizal fungi of plants have a negative impact on the cereal aphid *R. padi*, but positive effects on related plants and parasitoids [181]. Plant virus infection, on the other hand, can increase aphid size [182], as well as parasitoid larvae mortality and developmental times [183].

Intraguild predation by generalist predators has been found to disrupt the cereal aphid control exerted by primary parasitoids [160]. In contrast, other studies also found intraguild predation of predators on parasitoids in cotton aphids and soybean aphids, but in this case the intraguild predation seemed not to disrupt the aphid biological control [184,185]. Additionally, parasitoids can respond to intraguild predation by avoiding chemical traces of predators [161]. Furthermore, the mutualism of aphids with tending ants has been found to affect aphid–primary parasitoid–hyperparasitoid food webs, mainly by reducing the generalist hyperparasitoids [186].

## 12. The Role of Secondary Endosymbionts

In addition to the factors discussed above, the presence of secondary endosymbionts in their hosts is expected to influence the community composition and biocontrol efficiency of cereal aphid parasitoids. Secondary or facultative endosymbionts of aphids are maternally transmitted bacteria that, in contrast to the primary or obligate endosymbiont *Buchnera aphidicola* Munson et al. 1991 (Enterobacterales: Erwiniaceae), are not strictly required for host survival. It was first demonstrated by Oliver et al. [187], in pea aphids, that two secondary endosymbionts, *Hamiltonella defensa* Moran et al. 2005 (Enterobacterales: Enterobacteriaceae) and *Serratia symbiotica* Moran et al. 2005 (Enterobacterales: Yersiniaceae) [188], increased resistance to the parasitoid *A. ervi*. Further research has shown that endosymbionts conferring resistance to parasitoids are widespread in many species of aphids [189,190,191,192,193] and that “defensive” symbionts may also provide protection against natural enemies other than parasitoids, such as entomopathogenic fungi [194,195,196]. The secondary endosymbionts of cereal aphids have been increasingly surveyed over recent years, and the knowledge available so far suggests that the “usual suspects” [197,198] are present. *H. defensa, S. symbiotica, Regiella insecticola* Moran et al. 2005 and *Rickettsia* sp. have been reported from *S. avenae* [25,199,200,201,202,203]. Interestingly, in addition to *H. defensa, R. insecticola,* and *Rickettsia* sp., Li et al. [204,205,206] detected and described a previously unknown secondary endosymbiont (SMLS) (Rickettsiales: Rickettsiaceae) in *Sitobion miscanthi* (Takahashi) (Hemiptera: Aphididae) [204,205,206], which was also found in *R. padi* [204]. Additional secondary symbionts reported from *R. padi* and *R. maidis* include *S. symbiotica, H. defensa, R. insecticola, Rickettsia* sp., *Spiroplasma* sp., *Arsenophonus* sp., and *Wolbachia* sp. [207,208]. Telesnicki et al. [209] found a high frequency of infection with *H. defensa* in Argentinean *M. dirhodum*, which was mainly a host for *R. insecticola* in Chile [210].

Considering that *H. defensa* is associated with resistance to parasitoids in several aphid species [211], it was surprising that four strains of *H. defensa* from *S. avenae* did not provide any protection against the parasitoids *A. ervi* and *E. plagiator* [199]. However, it is known that different strains of *H. defensa* vary in the strength of resistance they provide [212], that protection can be specific against certain parasitoid species or even certain genotypes of the same parasitoid [192,213,214,215,216,217], and that parasitoids can evolve counteradaptations to overcome symbiont-conferred resistance [218,219,220]. It would thus be premature to assume a general absence of *H. defensa*-conferred protection in *S. avenae* or other cereal aphids based on one negative experimental result. Indeed, some evidence for *H. defensa*-mediated resistance to cereal aphid parasitoids has since been accumulated [25,193], but it is by no means universal [221,222].

Could hyperparasitoids be affected by aphids’ endosymbionts as well? This has never been tested in cereal aphids, but it seems unlikely, because in symbiont-protected aphids, the development of primary parasitoids is often arrested very early [223]. On the other hand, defensive symbionts have the potential for upward cascading effects to the trophic level of hyperparasitoids, by reducing the abundance of primary parasitoids in the food web [224]. This can alter food web composition. A field experiment with a non-cereal aphid, *Aphis fabae*, not only showed a strong reduction in parasitism in *Hamiltonella*-protected aphids, but also showed a dramatic change in the community composition of primary and secondary parasitoids, because some primary parasitoids were virtually excluded from plots with aphids harboring *H. defensa*. It will, thus, be important to consider the secondary endosymbionts harbored by cereal aphids when studying the parasitoid communities they support [25].

## 13. Effect of Agricultural Practices and Environment

The abundance of cereal aphids in field crops is affected by biotic and abiotic conditions that are linked to the environment and the agricultural practices followed. Temperature and precipitation affect the abundance of certain cereal aphid species. For example, the population growth of the grain aphid *S. avenae* increased when the mean fall temperature increased, but when the mean spring temperature and precipitation increased, populations decreased [225]. Similarly, wheat aphid, *S. graminum* populations decreased when the mean winter temperature and total fall precipitation increased [225]. Furthermore, Thierry et al. [226] reported that high temperatures during spring and winter caused a reduction of the bird cherry-oat aphid *R. padi* numbers, whereas high temperatures during fall increased the abundance of *R. padi*. Increases of wind speed, relative humidity, and precipitation decreased the abundance of cereal aphids caught in suction traps [227]. In a recent study, high levels of CO_2_, which was used as a fertilizer, and high temperatures decreased *R. padi* development and fecundity [228]. Soil fertilization with inorganic fertilizer accelerated the development and increased the fecundity of *S. avenae*, when the infested wheat plants were sufficiently watered [229]. Furthermore, 0.4 NH_4_NO_3_ g/plant of four different types of nitrogen fertilizers, enhanced the fecundity and the weight of adults of *R. padi* and *S. avenae* in contrast with 0.1 or 0.2 NH_4_NO_3_ g/plant [230]. The population densities of *R. padi* and *M. dirhodum* were higher when the fertilization with nitrogen was high (80 kg N/ha as manure vs. 80 kg N/ha as manure + 110 kg N/ha as mineral fertilizer) [231]. In some cases, nitrogen was not a favorable fertilizer for cereal aphid growth, in contrast to phosphorus, which enhanced the aphid infestation [232]. In addition, a 69-46-25 kg/acre N-P-K fertilization reduced cereal aphid populations, while simultaneously increasing the yield of the crop [233]. Nitrogen fertilization with maize straw amendment caused a reduction in the number of the aphids [234]. Interestingly, fertilization can alter certain aphid characteristics. For example, nitrogen fertilization caused *R. padi* and *S. avenae* individuals to gain body weight [235].

Since cereal aphid populations can be controlled by beneficial organisms, their impact is highly affected by environmental conditions [236,237,238,239]. For instance, nitrogen fertilizer led to an increase of parasitism of *A. colemani* and *A. rhopalosiphi* on *S. avenae* and *R. padi,* respectively [239]. Furthermore, the abundance of *S. avenae, S. graminum,* and *R. padi*, as well as the parasitoid species *A. avenae*, *Praon rhopalosiphum* Takada, and *Aphelinus albipodus* Hayat and Fatima (Hymenoptera: Aphelinidae), were increased by landscape complexity [237]. Similarly, Plećaš et al. [13] documented that large non-crop habitats, with high landscape heterogeneity, increased the population of cereal aphids, their parasitoids, and hyperparasitoids. Winqvist et al. [240] documented that landscape homogeneity resulted in less predation on cereal aphids. The authors also found that predation in non-organic (conventional) fields was higher than in organic fields within simple landscapes. In a recent study, the abundance of *S. miscanthi* and *R. padi* was decreased when five different varieties of winter wheat were sown in a field [241]. Different wheat genotypes can also determine aphid abundance. Batyrshina et al. [242] reported that the tetraploid wild emmer *Triticum turgidum* ssp. *dicoccoides* cv. Zavitan was the most resistant among three varieties, while the hexaploid spring bread *Triticum aestivum* cultivar Rotem was the most susceptible. The authors documented that aphids did not colonize juvenile plant tissues, preferring the matured spikes and flag leaves; therefore, the aphid population was higher on plants that matured earlier. *R. padi* heavily colonized maize but not grasslands that surrounded wheat seedlings, indicating that neighboring landscapes aid or deteriorate aphid infestation [243]. However, not all neighboring plants are useful, since certain species enhance the presence of cereal aphids. For instance, flower strips in-between fields increased the abundance of cereal aphids [244]. However, wildflower strips enhanced aphid parasitism and predation [245].

Pesticide applications are of major importance, since their irrational use may cause the evolution of resistance in exposed aphids [246,247,248]. Due to the continuous use of pyrethroids [247,248], some *S. avenae* strains exhibit the L1014F (*kdr*) mutation that makes them resistant to these insecticides [249]. Similarly, Wang et al. [247] documented that a strain of *R. padi* resistant to lambda-cyhalothrin exhibited cross-resistance to another nine active compounds. On the other hand, some insecticides, such as imidacloprid, controlled *R. padi, S. graminum,* and *S. avenae* aphids, resulting in a yield enhancement [250]. The application of imidacloprid should be handled cautiously, since it does not only affect cereal aphids, but their parasitoids as well [251]. Furthermore, insecticides did not reduce aphid densities in the presence of their natural enemies, while in their absence, insecticides were effective [252]. Apart from aphids, insecticides also kill the aphids’ natural enemies, and therefore aphid populations resurge more quickly after insecticidal treatments [252]. The year of insecticidal treatments should also be taken into consideration, since insecticides can be more or less efficient due to the prevalent environmental conditions. For instance, an experiment that took place during 2008–2009 revealed that cereal aphid densities were not reduced by treatment with the pyrethroid insecticide lambda-cyhalothrin. However, when the same experiment was repeated during 2010–2011, the same insecticide killed the exposed aphids [253]. This fact could be attributed to the high precipitation levels occurring in 2008–2009 vs. 2010–2011 [253]. The efficacy of insecticides may differ among strains of the same aphid species. For instance, Zuo et al. [254] reported that twelve geographical strains of *R. padi* exhibited variable levels of susceptibility or resistance to ten insecticidal active ingredients. Recently, Cao et al. [255] documented that short exposures of *S. avenae* to higher temperatures altered the tolerance of this species to imidacloprid.

Overall, the effects of the environment and agricultural practices are multifarious and can strongly affect the biological traits of aphids and their associated natural enemies in cereal fields, consequently increasing or decreasing the yield of the infested crops. These parameters should be taken into account when insecticidal management strategies are employed against cereal aphids. The use of registered insecticides at label doses and the rotation of the active ingredients can be suggested as management strategies, to ensure delayed development of the insecticidal resistance of cereal aphids [247,254].

## 14. Landscape Complexity

As already mentioned above, the structure of a whole landscape can influence aphids and their parasitoids. A structurally complex landscape can improve cereal aphid abundance and the primary parasitism rate, by providing more overwintering sites, alternative hosts, food sources, and shelter after harvest [27,256]. Such positive effects were found in several studies [13,237], while no effect of landscape complexity was shown in other studies [24,166,257]. Additionally, such impacts of landscape structure can vary during the seasons [256]. Differences in the species diversity of primary parasitoids in simple and complex structured landscapes were observed [13] or not [26,138,139]. Higher diversity of cereal aphid parasitoids may lead to higher biological control, as different species invade fields at different times during the season to parasitize aphids (cp. section “Host location, specialization, and exploitation of aphid colonies” in this review).

Hyperparasitoids can also be affected by landscape structure, even more strongly than primary parasitoids [138,237,258]. Furthermore, it was shown that more hyperparasitoid species hatched from both stinging nettle aphids on the field margin and cereal aphids in the adjacent field than primary parasitoid species [138]. Derocles et al. [157] also found that aphids inside and outside of agricultural fields shared almost no primary parasitoid species. Therefore, hyperparasitoids can link different habitats and may gain more advantages from the provision of semi-natural habitats in agricultural landscapes than primary parasitoids [138], which may increase the effect of hyperparasitoids on primary parasitoids, due to hyperparasitoid individuals invading fields from surrounding semi-natural habitats.

Landscape complexity may also affect cereal aphid–primary parasitoid and primary parasitoid–hyperparasitoid food webs, as shown, for example, by the reduced complexity of such food webs in structurally rich landscapes compared to simple ones [24,139]. This shows that the effect of landscape structure can be variable and different at different trophic levels. This has to be taken into account when landscape structure is changed (e.g., by sowing flowering strips adjacent to fields) to enhance biological control of cereal aphids.

## 15. Conclusions

Aphids are one of the most important limiting factors of cereal production, by causing direct damage or by transmitting viral pathogens to cereal crops. Aphid parasitoids are diverse and effective natural enemies of cereal aphids. Surprisingly, the basic biological and taxonomic characteristics of species participating in the cereal aphid food webs are poorly known, including cereal aphids endosymbionts that potentially affect parasitoids and hyperparasitoids. Most existing studies have considered a few economically important aphid species (e.g., *S. avenae, R. padi,* and *M. dirhodum*) and a few parasitoid species (e.g., *A. ervi, A. rhopalosiphi,* and *P. volucre*), not considering the full complexity of cereal aphid food webs, including hyperparasitoids and endosymbionts. Predicting the stability and dynamics of cereal aphid food webs requires understanding the food web diversity of all belonging trophic members and their interactions. One of the most problematic issues for parasitoid food web research is the common phenomenon of cryptic speciation and the ensuing problems for taxonomy. The community composition of secondary endosymbionts and their role in the biocontrol efficiency of cereal aphid parasitoids are further important issues to consider in biocontrol strategies. Cereal aphid food webs are also contingent on the surrounding habitats and landscape complexity, which can also affect cereal aphids and their associated parasitoids in very different ways. Finally, a proper combination of insecticidal treatments with agricultural practices suited for the prevailing environmental conditions can lead to the optimization of cereal aphid management with minimum detrimental impacts on natural enemies and the environment. Although we tried to integrate fundamental research in the ecology, taxonomy, and biodiversity of cereal aphid food webs and applied research about the effective control of cereal aphid pests, we are aware that this approach needs further justification from future research, especially in the context of climate change.

## Figures and Tables

**Table 1:** Cereal aphid parasitoids (Hymenoptera: Braconidae: Aphidiinae) in Europe.

Aphidiinae Species	Aphid Hosts	References
*Aclitus obscuripennis* Förster	*Anoecia* sp.	[9]
*Adialytus ambiguus* (Haliday)	*Sipha maydis* Passerini, *Sipha elegans* del Guercio	[10,43,44]
*Aphidius avenae* Haliday	*Metopolophium dirhodum* (Walker), *Rhopalosiphum padi* (L.), *Schizaphis graminum* (Rondani), *Sitobion avenae* (F.), *Sitobion fragariae* (Walker)	[9,10,44]
*Aphidius colemani* Viereck	*R. padi*, *S. avenae*	[9,10,44]
*Aphidius ervi* Haliday	*Diuraphis noxia* (Kurdjumov), *M. dirhodum*, *S. graminum*, *Rhopalosiphum maidis* (Fitch), *R. padi*, *S. avenae*, *S. fragariae*	[9,10,34,44]
*Aphidius matricariae* (Haliday)	*D. noxia*, *R. maidis*, *R. padi*, *S. graminum*, *S. avenae*, *S. fragariae*	[9,34,44,45]
*Aphidius rhopalosiphi* De Stefani	*D. noxia*, *M. dirhodum*, *Metopolophium festucae* (Theobald), *R. maidis*, *R. padi*, *S. graminum*, *S. avenae*, *S. fragariae*	[9,10,34,44]
*Aphidius uzbekistanicus* Luzhetzki	*M. dirhodum*, *M. festucae*, *S. avenae*, *S. fragariae*, *S. graminum*	[9,10,44,45]
*Binodoxys angelicae* (Haliday)	*S. avenae*	[9]
*Diaeretiella rapae* (M’Intosh)	*D. noxia*, *R. maidis*, *R. padi*, *S. avenae*, *S. graminum*	[9,10,34,44,45]
*Ephedrus plagiator* (Nees)	*Anoecia* sp., *M. dirhodum*, *R. maidis*, *R. padi*, *S. graminum*, *S. avenae*, *S. fragariae*	[9,10,44,45]
*Lipolexis gracilis* Förster	*R. padi*	[9]
*Lipolexis labialis* Tomanović and Kocić	*Anoecia corni* (F.)	[46]
*Lysiphlebus dissolutus* (Nees)	*A. corni*	[9]
*Lysiphlebus fabarum* (Marshall)	*R. maidis*, *S. avenae*	[9,10,44]
*Lysiphlebus testaceipes* (Cresson)	*R. maidis*, *S. graminum*, *R. padi*, *S. avenae*	[10,34,44]
*Monoctonus caricis* (Haliday)	*R. padi*, *Sitobion* sp.	[47]
*Paralipsis brachycaudi* Tomanović and Starý	*Tetraneura ulmi* (L.)	[48]
*Paralipsis enervis* (Nees)	*Geoica utricularia* (Passerini)	[48]
*Praon abjectum* (Haliday)	*R. padi*	[9]
*Praon gallicum* Starý	*M. dirhodum*, *R. padi*, *S. graminum*, *S. avenae*	[9,10,44]
*Praon necans* Mackauer	*R. padi*	[9]
*Praon volucre* (Haliday)	*M. dirhodum*, *R. padi*, *S. graminum*, *S. avenae*, *S. fragariae*	[9,10,44,45]
*Toxares deltiger* (Haliday)	*M. dirhodum*, *S. avenae*	[9,31]
*Trioxys auctus* (Haliday)	*R. padi*	[9,49]
*Trioxys sunnysidensis* Fulbright and Pike	*R. padi*	[50]

**Table 2:** Literature review of hyperparasitoids of cereal aphid parasitoid in European agroecosystems. Kend denotes koinobiont endohyperparasitoids. Iect denotes idiobiont ectohyperparasitoids.

Family	Hyperparasitoid Species	Aphids Hosting Parasitized Aphidiines	Biology	References
Encyrtidae	*Syrphophagus aphidivorus* (Mayr)	*M. dirhodum*, *R. padi*, *Rhopalosiphum* sp., *S. graminum*, *S. avenae*	Kend	[12,37,51]
	*Syrphophagus mamitus* (Walker)		Kend	[49]
Figitidae	*Alloxysta arcuata* (Kieffer)	*R. padi*	Kend	[52]
	*Alloxysta brachyptera* (Hartig)	*S. avenae*	Kend	[51]
	*Alloxysta brevis* (Thomson)	*M. dirhodum*, *R. maidis*, *R. padi*, *S. avenae*, *Sipha* spp.	Kend	[12,37,51,52]
	*Alloxysta castanea* (Hartig)	*S. avenae, Hyalopterus pruni* (Geoffroy), *Hyalopterus* sp.	Kend	[53,52]
	*Alloxysta fracticornis* (Thomson)	*S. avenae*	Kend	[52]
	*Alloxysta fulviceps* (Curtis)		Kend	[37]
	*Alloxysta kovilovica* Ferrer-Suay and Pujade-Villar	*S. avenae*	Kend	[52]
	*Alloxysta leunisii* (Hartig)		Kend	[49]
	*Alloxysta macrophadna* (Hartig)		Kend	[49]
	*Alloxysta mullensis* (Cameron)	*Sipha* sp., *S. avenae*	Kend	[53,52]
	*Alloxysta pedestris* (Curtis)		Kend	[37]
	*Alloxysta victrix* (Westwood)	*M. dirhodum*, *R. padi*, *Rhopalosiphum* sp., *S. graminum*, *S. avenae, Sitobion* sp.	Kend	[12,37,51,53]
	*Phaenoglyphis villosa* (Hartig)	*M. dirhodum*, *R. padi*, *Rhopalosiphum* sp., *S. avenae,* *H. pruni, Hyalopterus* sp.	Kend	[12,37,51,53,54]
Megaspillidae	*Dendrocerus aphidum* (Rondani)	*S. avenae*	Iect	[51,55]
	*Dendrocerus carpenteri* (Curtis)	*M. dirhodum*, *R. padi*, *Rhopalosiphum* sp., *S. graminum*, *Sipha* sp., *S. avenae*	Iect	[12,51,55]
	*Dendrocerus laticeps* (Hedicke)		Iect	[49]
	*Dendrocerus rectangularis* (Kieffer)		Iect	[49]
	*Dendrocerus serricornis* (Boheman)	*S. avenae*	Iect	[55]
Pteromalidae	*Asaphes suspensus* (Nees)	*M. dirhodum*, *R. padi*, *Rhopalosiphum* sp., *S. graminum*, *S. avenae*	Iect	[12,51]
	*Asaphes vulgaris* Walker	*M. dirhodum*, *Rhopalosiphum* sp., *S. avenae*	Iect	[12,37,51]
	*Coruna clavata* Walker	*S. avenae*	Iect	[37,49]
	*Pachyneuron aphidis* (Bouché)	*S. avenae*, *M. dirhodum*	Iect	[12,37]
	*Pachyneuron concolor* (Förster)	*M. dirhodum*, *S. avenae*	Iect	[12]
	*Pachyneuron formosum* Walker	*S. avenae*	Iect	[37,49]
	*Pachyneuron muscarum* (L.)	*R. maidis*, *S. avenae*	Iect	[37,51]
	*Pachyneuron solitarium* (Hartig)	*S. avenae*	Iect	[37]

## Data Availability

Data are contained within the article.
